# Design and Analysis of a Turning Dynamometer Embedded in Thin-Film Sensor

**DOI:** 10.3390/mi10030210

**Published:** 2019-03-26

**Authors:** Yuntao Zhang, Wenge Wu, Yanwen Han, Haijun Wen, Yunping Cheng, Lijuan Liu

**Affiliations:** School of Mechanical Engineering, North University of China, Taiyuan 030051, Shanxi, China; zyt7262@126.com (Y.Z.); hyw9226@126.com (Y.H.); wenhaijun@nuc.edu.cn (H.W.); ypchengbk@163.com (Y.C.); liulijuan@nuc.edu.cn (L.L.)

**Keywords:** dynamometer, film sensor, structural dimensions, strain sensitivity coefficient

## Abstract

This paper proposes a high-strain sensitivity turning dynamometer that combines several thin-film resistor grids into three Wheatstone full-bridge circuits that can measure triaxial cutting forces. This dynamometer can replace different cutter heads using flange connections. In order to improve the strain effect of the dynamometer, the strain film sensor is fixed on the regular octagonal connection plates on both ends of the elastomer by vacuum brazing, and the stepped groove structure is also designed inside the elastomer. The dynamometer model is simplified as a four-segment cantilever beam which has different sections. The measurement mechanism model of the dynamometer system is established by the transformation relationship between deflection and strain, under external force. The standard turning tool of 20 mm square is used as a reference. The influence of the structural dimensions of the dynamometer on its strain sensitivity coefficient *K* is studied. The applicability of the theoretical model of dynamometer strain is verified by finite element analysis. Finally, the dynamometer with the largest *K* value is subjected to the bending test and compared with a standard turning tool. The experimental results show that the measurement sensitivity of the dynamometer is 2.32 times greater than that of the standard turning tool. The results also show that this dynamometer can effectively avoid the influence of the pasting process on strain transmission, thus indicating its great potential for measuring cutting force in the future.

## 1. Introduction

As the development of the micromachine field increases rapidly, the requirements for machining accuracy in machine manufacturing are also increasing. The cutting force is an extremely important physical parameter in the metal cutting process. The state of the machining workpiece, such as the quality of the workpiece and the power consumption of the machine tool, can be directly judged by the cutting force. By studying the cutting force, the machining conditions can be improved and optimized to improve the accuracy of the workpiece. Therefore, the accurate measurement of cutting force is particularly important. In recent years, many researchers have devoted themselves to the miniaturization of dynamometers. They have combined sensing elements, tool holders, and turning tools to measure cutting forces [1,2,3,4]. Zhao, et al. proposed a sensor model for measuring triaxial cutting forces [5,6]. They used two mutually perpendicular octagonal ring structures as elastic elements. Their experiments showed that the cutting force sensor combined with the structure had good accuracy with an error from 0.38% to 0.83%, and they found through modal analysis that the cutting force sensor also had a superior natural frequency. Richárd Horváth and Tibor Pálinkás designed a dynamometer for measuring cutting forces in fine cutting [7]. They installed a piezoelectric cell under the cutting insert which had a new tool holder structure. The cutting force was measured by the electrical charge generated by the mechanical deformation of the piezoelectric crystal. Their experiments showed that the measuring accuracy of the dynamometer was 0.1 N. However, these researchers did not reveal the measurement mechanism of the dynamometer, and they did not consider the influence of the structural parameters of the dynamometer on its own strength, stiffness, and sensitivity. Moreover, due to the limitation of the pasting process, the resistance strain gauge had a large measurement bias signal. The piezoelectric crystal [8,9,10,11] had a poor unidirectionality of the charge, so there was a serious interference phenomenon when the force was measured in three directions. In recent years, with the development of MEMS technology [12,13,14,15], various thin-film sensors have been widely used [16,17,18]. Tao, et al. embedded a thermocouple film into the cutting insert to measure the cutting heat [19]. Welf-Guntram Drossel, et al. prepared a piezoelectric film sensor by chemical vapor deposition in order to measure the cutting force during milling [20].

The design proposed is a turning dynamometer embedded with a Ni–Cr alloy film strain sensor by taking advantage of the low temperature coefficient and high-strain sensitivity coefficients of the Ni–Cr alloy film [21,22]. This dynamometer eliminates the coupling effect of multi-directional forces and enables accurate measurement of each unidirectional force. The mechanism reveals the influence of the structural parameters of the dynamometer on the measurement sensitivity of the system. Experiments show that the dynamometer has a high-strain sensitivity coefficient and good linearity, revealing its great potential for measuring cutting force. In addition to this, the paper outlines a solid theoretical foundation for subsequent product application.

## 2. Dynamometer Structure Model and Measurement Principle

**Figure 1:**
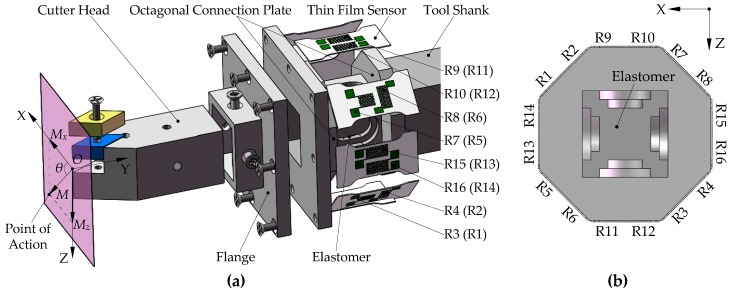
Dynamometer model structure: (**a**) Explosion view of the dynamometer structure, (**b**) mutual positional relationship of resistance grids from *R*_1_ to *R*_16_.

The turning dynamometer with an embedded film sensor consists of a replaceable cutter head, flange, elastomer, octagonal connection plate, strain film sensor, and tool shank, as shown in Figure 1. In order to improve the strain sensitivity of the dynamometer, the surfaces of the four sides of the elastomer are designed with stepped grooves, and at both ends of the elastomer the distance between the strain resistance grid and the neutral layer is increased by the octagonal connection plate. The cutter head is inserted into the elastomer through the flange to transmit the cutting force to the elastomer. Both ends of the film sensor are combined with the octagonal connection plate by vacuum brazing in order to eliminate the large impact of the pasting process on the strain transmission. The cutter heads with different angle shapes can be replaced on the dynamometer to achieve accurate measurements of the triaxial cutting force.

**Figure 2:**
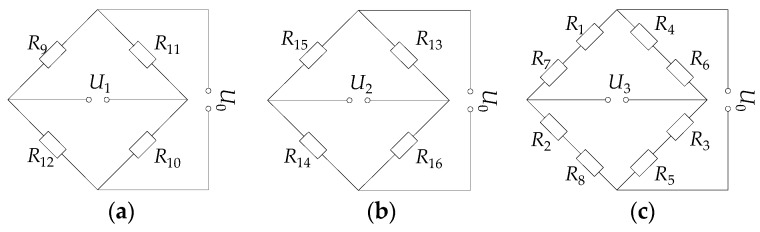
Wheatstone bridge circuit diagram: (**a**) Circuit 1, (**b**) circuit 2, (**c**) circuit 3.

As shown in Figure 1, the eight sensor units are mounted around the elastomer. The 16 thin-film resistor grids are connected into three Wheatstone bridge circuits, as shown in Figure 2. The bridge circuits 1–3 measure the main cutting force *F_z_*, the feeding force *F_x,_* and the thrust force *F_y_*, respectively, and their output voltages are:

(1) $$U_{1}=\frac{1}{4}\left(\frac{\Delta R_{9}}{R_{9}}+\frac{\Delta R_{10}}{R_{10}}-\frac{\Delta R_{11}}{R_{11}}-\frac{\Delta R_{12}}{R_{12}}\right)U_{0}$$

(2) $$U_{2}=\frac{1}{4}\left(\frac{\Delta R_{15}}{R_{15}}+\frac{\Delta R_{16}}{R_{16}}-\frac{\Delta R_{13}}{R_{13}}-\frac{\Delta R_{14}}{R_{14}}\right)U_{0}$$

(3) $$U_{3}=\frac{1}{4}\left[\left(\frac{\Delta R_{1}}{R_{1}}+\frac{\Delta R_{7}}{R_{7}}\right)+\left(\frac{\Delta R_{3}}{R_{3}}+\frac{\Delta R_{5}}{R_{5}}\right)-\left(\frac{\Delta R_{4}}{R_{4}}+\frac{\Delta R_{6}}{R_{6}}\right)-\left(\frac{\Delta R_{2}}{R_{2}}+\frac{\Delta R_{8}}{R_{8}}\right)\right]U_{0}$$

where *U*_0_ is the input voltage of the circuit. *R*_1_ to *R*_16_ are the initial resistance values of the 16 resistor grids, and they are equal to *R*. ∆*R*_1_ to ∆*R*_16_ are the changes in the resistance of each resistor grid in the circuit.

The coordinate origin *O* shown in Figure 1 is located in a plane perpendicular to the central axis of the dynamometer model and the plane passes the tool nose, also the origin *O* is collinear with the central axis. When the cutting force action point is at the *O* point, *F_y_* will exert an extrusion effect on the resistance grids from *R*_9_ to *R*_12_, and the resistance changes are the same; *F_z_* will exert tensile and compression effects on the resistance grids *R*_9_, *R*_10,_ *R*_11_, and *R*_12_ respectively. At this time, ∆*R*_9_ = ∆*R*_10_ = −∆*R*_11_ = −∆*R*_12_, the Equation (1) can be rewritten as:

(4) $$U_{1}=\frac{\Delta R_{\alpha }}{R}U_{0}=k_{\alpha }\varepsilon_{\alpha }U_{0}$$

where $\Delta R_{\alpha }$ is the resistance change of each resistor in circuit 1. $k_{\alpha }$ is the resistance strain coefficient of the resistance grids from *R*_9_ to *R*_12_. $\varepsilon_{\alpha }$ is the strain value of the resistance grids from *R*_9_ to *R*_12_.

Because the structure of the dynamometer model is symmetrical, Equation (2) can be rewritten as:

(5) $$U_{2}=\frac{\Delta R_{\beta }}{R}U_{0}=k_{\beta }\varepsilon_{\beta }U_{0}$$

where $\Delta R_{\beta }$ is the resistance change of each resistor in circuit 2. $k_{\beta }$ is the resistance strain coefficient of the resistance grids from *R*_13_ to *R*_16_. $\varepsilon_{\beta }$ is the strain value of the resistance grids from *R*_13_ to *R*_16_.

When the dynamometer is subjected to the binding force of *F_z_* and *F_x_*, ∆*R*_1_ = −∆*R*_3_, ∆*R*_2_ = −∆*R*_4_, ∆*R*_5_ = −∆*R*_7_, ∆*R*_6_ = −∆*R*_8_. At this time, *U*_3_ = 0 can be obtained from Equation (3). In other words, bridge circuit 3 can decouple *F_z_* and *F_x_*. However, the *F_y_* exerts extrusion effects on all the sensor units. The resistance values of the resistance grids *R*_1_, *R*_3_, *R*_5_, and *R*_7_ are equally decreased, and the resistance values of the resistance grids *R*_2_, *R*_4_, *R*_6_, and *R*_8_ are equally increased. Then, Equation (3) can be rewritten as:

(6) $$U_{3}=\left(\frac{\Delta R_{\gamma }}{R}+\frac{\Delta R_{\delta }}{R}\right)U_{0}=(k_{\gamma }+k_{\delta })\varepsilon_{\gamma }U_{0}$$

where $\Delta R_{\gamma }$ is the resistance change of *R*_1_, *R*_3_, *R*_5,_ and *R*_7_ in circuit 3. $\Delta R_{\delta }$ is the resistance change of *R*_2_, *R*_4_, *R*_6,_ and *R*_8_ in circuit 3. $k_{\gamma }$ and $k_{\delta }$ are the resistance strain coefficients of the two resistor grids arranged perpendicularly to each other. $\varepsilon_{\gamma }$ is the strain value of the resistance grids from *R*_1_ to *R*_8_.

When the cutting force action point is at any position, *F_z_* and *F_x_* will exert additional torque on the dynamometer model. Since the mounting positions of the eight sensors are symmetrical about the center of the structure, the full-bridge Wheatstone circuit not only eliminates the effects of cutting heat, but also decouples the torque action. However, the *F_y_* will exert additional bending moment *M* on the dynamometer model, which is decomposed into *M_x_* along the *X* direction and *M_z_* along the *Z* direction, as shown in the plane of Figure 1. The bending moment will couple the output results of the bridge circuits 1 and 2. At this time, the strain of the resistance grids from *R*_9_ to *R*_12_ depends on the joint action of *F_z_* and *M_z_*, and the strain of the resistance grids from *R*_13_ to *R*_16_ depends on the joint action of *F_x_* and *M_x_*.

## 3. Establishment of the Measurement Mechanism Model of Dynamometer

**Figure 3:**
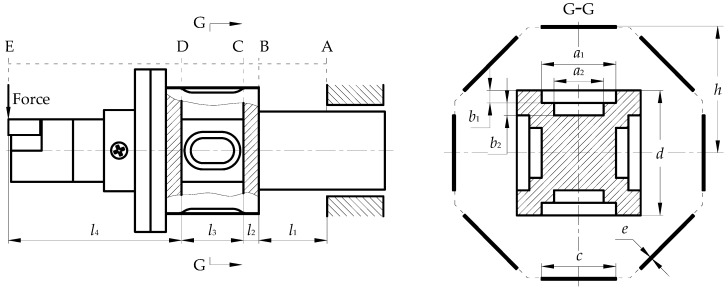
Dynamometer structure dimensions parameter.

Here, the model is equivalent to a cantilever beam for mechanical analysis and reveals the measurement mechanism of the dynamometer system. As shown in Figure 3, the approximate differential equation of the deflection crankshaft of the equal section cantilever beam [23] is:

(7) $$\frac{d^{2}\omega }{dx^{2}}=\frac{M_{\left(x\right)}}{EI}$$

Equation (7) is successively integrated twice to obtain:

(8) $$\left\{ \begin{array}{l} \varphi =\frac{d\omega }{dx}=\int \frac{M_{\left(x\right)}}{EI}dx+C\\ \omega =\int \int \frac{M_{\left(x\right)}}{EI}dxdx+Cx+D \end{array}\right.$$

where *φ* and *ω* are the rotation angle and the displacement of the cantilever beam, respectively, and $M_{\left(x\right)}$ is the bending moment of the corresponding section. Since the fixed end of the cantilever beam is completely constrained, when *x* = 0, then *φ* = 0, and *ω* = 0. Then the maximum angle and the displacement of the free end subjected to the constant force *F* are:

(9) $$\left\{ \begin{array}{l} \varphi_{\max1}=\frac{Fl^{2}}{2EI}\\ \omega_{\max1}=\frac{Fl^{3}}{3EI} \end{array}\right.$$

The maximum angle and displacement where the free end are subjected to the bending moment $M_{e}$ are:

(10) $$\left\{ \begin{array}{l} \varphi_{\max2}=\frac{M_{e}l}{EI}\\ \omega_{\max2}=\frac{M_{e}l^{2}}{2EI} \end{array}\right.$$

In the above formula, *l*, *E*, and *I* are the length, elastic modulus, and moment of inertia of the cantilever beam of equal section, respectively. For the model shown in Figure 3, segmentation processing is required. First, the BE segment is rigidified, and the angle and displacement of the AB segment are obtained:

(11) $$\left\{ \begin{array}{l} \varphi_{B}=\frac{Fl_{1}{}^{2}}{2EI_{1}}+\frac{Fl_{1}\left(l_{2}+l_{3}+l_{4}\right)}{EI_{1}}\\ \omega_{B}=\frac{Fl_{1}{}^{3}}{3EI_{1}}+\frac{Fl_{1}{}^{2}\left(l_{2}+l_{3}+l_{4}\right)}{2EI_{1}} \end{array}\right.$$

At this time, the influence of the deformation of the AB section on the D section is:

(12) $$\left\{ \begin{array}{l} \varphi_{D1}=\varphi_{B}\\ \omega_{D1}=\omega_{B}+\varphi_{B}\left(l_{2}+l_{3}\right) \end{array}\right.$$

The influence of the deformation of the AB section on the C section is:

(13) $$\left\{ \begin{array}{l} \varphi_{C1}=\varphi_{B}\\ \omega_{C1}=\omega_{B}+\varphi_{B}l_{2} \end{array}\right.$$

Secondly, the AB segment and the CE segment are rigidized, and the corner and displacement of the BC segment are obtained:

(14) $$\left\{ \begin{array}{l} \varphi_{C2}=\frac{Fl_{2}{}^{2}}{2EI_{2}}+\frac{Fl_{2}\left(l_{3}+l_{4}\right)}{EI_{2}}\\ \omega_{C2}=\frac{Fl_{2}{}^{3}}{3EI_{2}}+\frac{Fl_{2}{}^{2}\left(l_{3}+l_{4}\right)}{2EI_{2}} \end{array}\right.$$

At this time, the influence of the BC section deformation on the D section is:

(15) $$\left\{ \begin{array}{l} \varphi_{D2}=\varphi_{C2}\\ \omega_{D2}=\omega_{C2}+\varphi_{C2}l_{3} \end{array}\right.$$

Finally, the AC and DE segments are rigidized, and the corners and displacements of the CD segment are obtained:

(16) $$\left\{ \begin{array}{l} \varphi_{D3}=\frac{Fl_{3}{}^{2}}{2EI_{3}}+\frac{Fl_{3}l_{4}}{EI_{3}}\\ \omega_{D3}=\frac{Fl_{3}{}^{3}}{3EI_{3}}+\frac{Fl_{3}{}^{2}l_{4}}{2EI_{3}} \end{array}\right.$$

The deformation of each segment can be superimposed to obtain the total displacement of the C section and the D section under the action of the main cutting force *F_z_*:

(17) $$\left\{ \begin{array}{l} \omega_{C}=\omega_{C1}+\omega_{C2}\\ \omega_{D}=\omega_{D1}+\omega_{D2}+\omega_{D3} \end{array}\right.$$

Assuming that the C section is completely constrained, the CD segment is considered to be an equal section cantilever beam model, and the relative displacement of the free end of D section is:

(18) $$\omega_{DC-Z}=\omega_{D}-\omega_{C}=F_{z}\left(\frac{l_{1}^{2}l_{3}+2l_{1}l_{3}(l_{2}+l_{3}+l_{4})}{2EI_{1}}+\frac{l_{2}^{2}l_{3}+2l_{2}l_{3}(l_{3}+l_{4})}{2EI_{2}}+\frac{2l_{3}{}^{3}+3l_{3}{}^{2}l_{4}}{6EI_{3}}\right)$$

Due to the symmetry of the dynamometer model structure, the relative displacement of the feeding force *F_x_* to the D section can also be calculated as:

(19) $$\omega_{DC-X}=F_{x}\left(\frac{l_{1}^{2}l_{3}+2l_{1}l_{3}(l_{2}+l_{3}+l_{4})}{2EI_{1}}+\frac{l_{2}^{2}l_{3}+2l_{2}l_{3}(l_{3}+l_{4})}{2EI_{2}}+\frac{2l_{3}{}^{3}+3l_{3}{}^{2}l_{4}}{6EI_{3}}\right)$$

By the same Equation (10), the relative moment of the D section along the *X* and *Z* directions in the dynamometer model can be obtained by the bending moment generated by *F_y_*:

(20) $$\left\{ \begin{array}{l} \omega_{Y-X}=F_{y}s\left(\frac{l_{1}l_{3}}{EI_{1}}+\frac{l_{2}l_{3}}{EI_{2}}+\frac{l_{3}{}^{2}}{2EI_{3}}\right)\cos\theta \\ \omega_{Y-Z}=F_{y}s\left(\frac{l_{1}l_{3}}{EI_{1}}+\frac{l_{2}l_{3}}{EI_{2}}+\frac{l_{3}{}^{2}}{2EI_{3}}\right)\sin\theta \end{array}\right.$$

The expression of $\varepsilon_{\alpha }$ and $\varepsilon_{\beta }$ in the measurement system is obtained from the relationship between strain and deflection in material mechanics [23]:

(21) $$\left\{ \begin{array}{l} \varepsilon_{\alpha }=\frac{3hI_{3}}{4l_{3}^{2}I_{0}}(\omega_{DC-Z}+\omega_{Y-Z})\\ \varepsilon_{\beta }=\frac{3hI_{3}}{4l_{3}^{2}I_{0}}(\omega_{DC-X}+\omega_{Y-X}) \end{array}\right.$$

where *E* is the elastic modulus of the sensor, *I*_0_ is the moment of inertia of the dynamometer at the center of the resistance grid for the neutral layer, and its expression [23] is Equation (22). *I_i_* and *l_i_* (*i* = 1, 2, 3) are the initial moment of inertia and length of each segment of the dynamometer model, and *h* is the distance from the sensor to the neutral layer. In addition, *s* is the distance from the point of action of the cutting force in the plane of Figure 1 to the coordinate origin *O*, and *θ* is the angle from the counterclockwise direction to the direction of the bending moment of the *x*-axis in the plane of Figure 1.

(22) $$I_{0}=\frac{d^{4}}{12}-\sum_{i=1}^{n}\left[\frac{a_{i}b_{i}^{3}+b_{i}a_{i}^{3}}{6}+2a_{i}b_{i}{\left(\frac{d-b_{n}}{2}-\sum_{i=1}^{n}b_{i-1}\right)}^{2}\right]+2{\left(h-\frac{e}{2}\right)}^{2}ce+2ceh^{2}+\frac{ce^{3}+ec^{3}}{6}+\frac{\sqrt{2}ec^{3}}{12}$$

where *c* and *e* represent the width and thickness of the sensor at the G-G section, respectively, and *d* represents the side length of the *y*-axial section of the elastomer. In addition, *a_i_* and *b_i_* (*i* = 1,2...,*n*) represent the width and depth of the section groove, respectively.

The output of the bridge circuit 3 mainly depends on the extrusion effect of the *F_y_* on the dynamometer, so $\varepsilon_{\gamma }$ can be expressed as:

(23) $$\varepsilon_{\gamma }=\frac{\sigma_{\gamma }}{E}=\frac{F_{y}}{AE}$$

where *A* is the area of the radial section of the dynamometer which is the location of the sensor.

By integrating the above formulas, the relationship between the output voltages of the three bridge circuits and the cutting force components in the measurement system proposed in this paper can be obtained:

(24) $$\left\{ \begin{array}{l} U_{1}=(K_{1}F_{z}+K_{2}s\sin\theta F_{y})k_{\alpha }U_{0}\\ U_{2}=(K_{1}F_{x}+K_{2}s\cos\theta F_{y})k_{\beta }U_{0}\\ U_{3}=K_{3}F_{y}(k_{\gamma }+k_{\delta })U_{0} \end{array}\right.$$

where *K*_1_–*K*_3_ are the strain sensitivity coefficients of the dynamometer. The relationship with the structural parameters of the dynamometer is shown in Equation (25). Equation (24) demonstrates that the output signal of the measurement system will be affected after the cutter head is replaced in the dynamometer. Therefore, the parameters of the system measurement mechanism need to be reset each time the cutter head is replaced in order to ensure the reliability of the system output.

(25) $$\left\{ \begin{array}{l} K_{1}=\frac{3hI_{3}}{4l_{3}^{2}I_{0}}\left(\frac{l_{1}^{2}l_{3}+2l_{1}l_{3}(l_{2}+l_{3}+l_{4})}{2EI_{1}}+\frac{l_{2}^{2}l_{3}+2l_{2}l_{3}(l_{3}+l_{4})}{2EI_{2}}+\frac{2l_{3}{}^{3}+3l_{3}{}^{2}l_{4}}{6EI_{3}}\right)\\ K_{2}=\frac{3hI_{3}}{4l_{3}^{2}I_{0}}\left(\frac{l_{1}l_{3}}{EI_{1}}+\frac{l_{2}l_{3}}{EI_{2}}+\frac{l_{3}{}^{2}}{2EI_{3}}\right)\\ K_{3}=\frac{1}{AE} \end{array}\right.$$

where *I*_2_ = 0.8758*h*^4^. It is obvious from Equation (25) that the dynamometer structure dimensions are especially important for the system’s measurement sensitivity. If the length of *l*_3_ is increased, then *K*_1_ and *K*_2_ are decreased. If the length of *l*_1_, *l*_2_ and *l*_4_ are increased, then *K*_1_ and *K*_2_ are increased. When the *K* value is increased, improvement in the segments with a smaller moment of inertia are given priority. Therefore, a preference should be taken to increase the length of *l*_1_ and *l*_4_ when the dynamometer is designed. This guidance is significance for improving the measurement sensitivity of the dynamometer after calibrating the force range.

## 4. Fabrication and Calibration of Thin-Film Sensors

**Figure 4:**
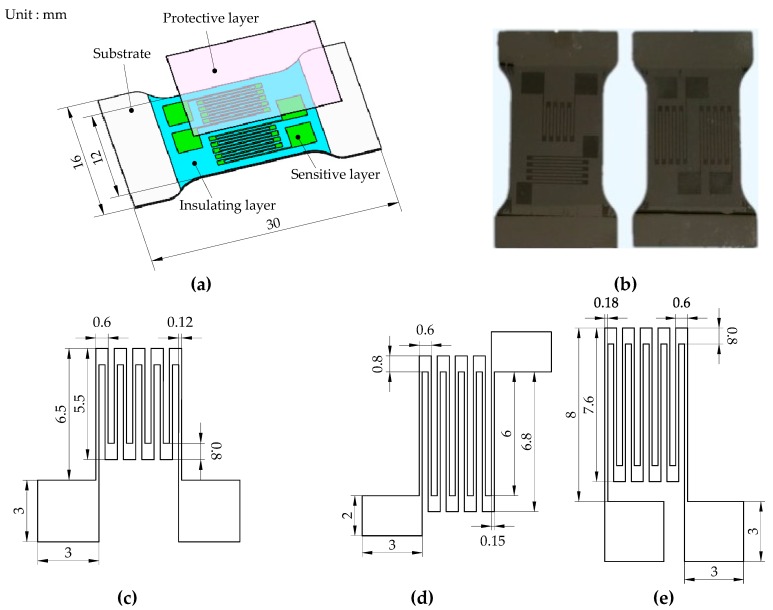
Alloy film sensor: (**a**) Sensor structure, (**b**) fabricated sensor, (**c**) sizes of the resistance grids *R*_1_, *R*_3_, *R*_5_, and *R*_7_, (**d**) sizes of the resistance grids *R*_2_, *R*_4_, *R*_6_, and *R*_8_, (**e**) sizes of the resistance grids from *R*_9_ to *R*_16_.

The alloy thin-film sensor is mainly fabricated using a FJL-560a magnetic control and ion beam composite sputter deposition system (SKY Technology Development Co., Ltd., CAS, Shenyang, China) and an EVG610 double-sided lithography machine (EV Group, Schärding, Austria). The fabrication parameters and basic characteristics of the film are shown in Table 1. The thin-film sensor is composed of stainless steel substrate, a Si_3_N_4_ insulating layer, a Ni_80_Cr_20_ sensitive layer, and a Si_3_N_4_ protective layer. In order to insulate the sensor substrate from the sensitive layer, a thicker Si_3_N_4_ film is first deposited on the substrate. Then, a Ni_80_Cr_20_ film is deposited as a sensor sensitive layer, and resistance grids and electrodes are formed by photolithography. In order to prevent the sensitive layer from being oxidized and etched, a protective layer of Si_3_N_4_ film is finally deposited on the surface of the sensor, as shown in Figure 4.

Reference [24] indicates that the actual measured value of the thin-film resistance grid is about five times the theoretical design, and the ratio of the two may increase by more than 10 times due to the influence of the film roughness. Therefore, the theoretical resistance of each of the resistance grids is designed to be 500 Ω, and the sizes of the resistance grids from *R*_1_ to *R*_16_ are as shown in Figure 4. The actual resistance of the resistance grid is between 1821 Ω and 2216 Ω determined by measuring the resistance of the sensor. Compared with the expected resistance of 2000 Ω, the maximum error is 10.8%. According to the layout position of the resistance grids from *R*_1_ to *R*_16_ in the turning dynamometer, several film sensors within 1% of the resistance error are selected to be installed in the dynamometer.

**Figure 5:**
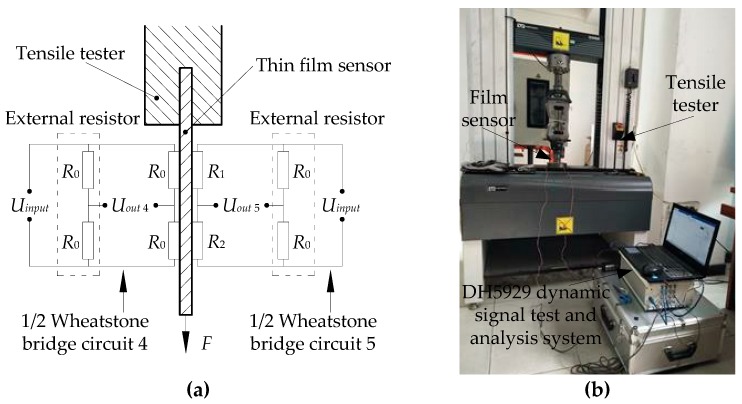
Film sensor uniaxial tensile test: (**a**) Bridge circuit diagram, (**b**) experimental device.

**Figure 6:**
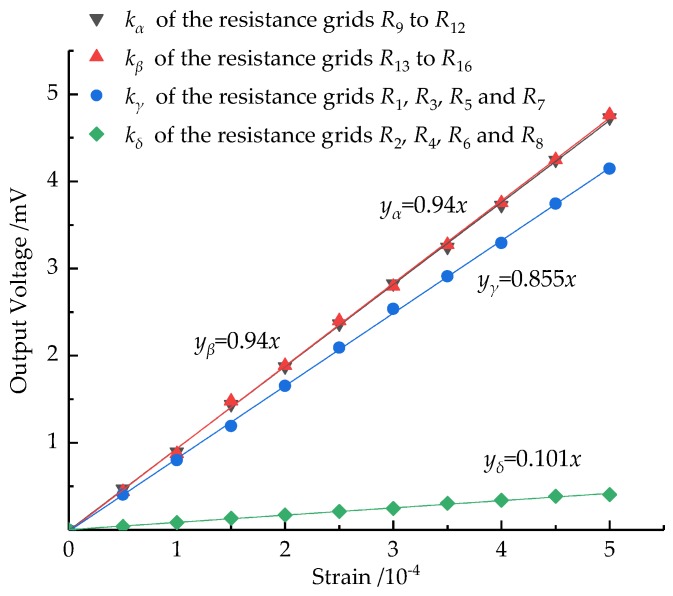
Calibration signal of the thin-film sensor.

The strain sensitivity coefficient of each film sensor is calibrated using the uniaxial tensile test. The test device mainly includes a DH5929 dynamic signal test (Instron, Shanghai, China) and an analysis system, a film sensor and a tensile tester, as shown in Figure 5. Two resistance strain gauges are attached to the back surface of the substrate at the same position as the sensor resistance grids. The two strain gauges are connected to the external resistors as the one-half bridge circuit 4. *R*_1_ and *R*_2_ are thin-film resistance grids in the sensor. *R*_1_ and *R*_2_ are connected to two external resistors as the one-half bridge circuit 5. There is an input voltage of 10 V in circuit 4 and circuit 5, respectively. The unidirectional tensile force is applied to the film sensor by the tensile tester in the elastic strain range. The strain of the elastic substrate is measured using circuit 4. Then, the relationship curves in Figure 6 are obtained by measuring the output voltage of circuit 5. Among them, the fitting function of these curves is:

(26) $$U_{out}=\frac{1}{2}k\varepsilon U_{in}$$

where *U_in_* is the input voltage of the circuit. *k* is the resistance strain coefficient of the thin-film resistor grid. *ε* is the strain of the thin-film resistor grid, which is equivalent to the strain of the elastic substrate. *U_out_* is the output voltage of circuit 5.

Figure 6 shows that the thin-film sensor has good linearity when converting strain into electrical signals, which is extremely important for the measurement of the cutting force. In the Wheatstone half-bridge circuit, the relationship curve slope of the sensor output voltage and its strain is 0.5*k*. From the curve in Figure 6, the resistance strain coefficients of the film resistor grids $k_{\alpha }$,$k_{\beta }$,$k_{\gamma }$ and $k_{\delta }$ are 1.88, 1.88, 1.71, and 0.26, respectively. Generally, the resistance strain coefficient of the nickel-chromium film is approximately 1–2, which indicates that the film sensor can better convert the strain of the substrate into the strain of the film sensitive grid along the direction of the applied force.

## 5. Dynamometer Bending Test and Simulation

**Figure 7:**
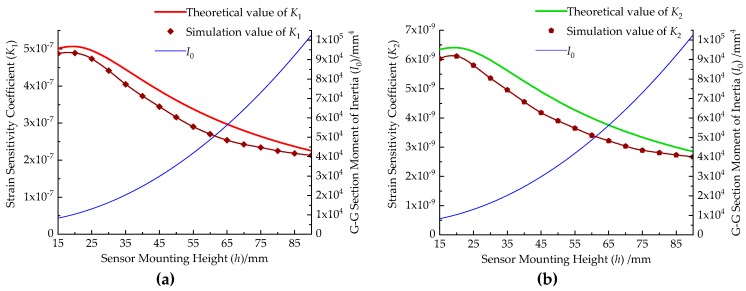
Relationship between sensor mounting height and dynamometer strain sensitivity coefficient: (**a**) *K*_1_ and *h* relationship curve, (**b**) *K*_2_ and *h* relationship curve.

The standard turning tool of 20 mm square is used as a reference. Here, the structural dimensions of the dynamometer are selected as *l*_1_ = 20 mm, *l*_2_ = 5 mm, *l*_3_ = 20 mm, *l*_4_ = 56 mm, and *d* = 20 mm. Under the condition of ensuring the strength and rigidity of the dynamometer, the second step groove is designed in the elastomer, and takes *a*_1_ = 12 mm, *a*_2_ = 8 mm, *b*_1_ = *b*_2_ = 2 mm. Then, this dynamometer is simulated using ABAQUS software (Dassault Simulia, Providence, RI, USA). Due to the space limitation of the sensor’s regular octagonal arrangement, *h* ≥ 14.14 mm is necessary. In the range of the sensor mounting height from 15 mm to 90 mm, either *F_z_* of 1000 N or *M_z_* of 10 N·m is applied every 5 mm to the tool nose of the dynamometer. Finally, the strain sensitivity coefficient of the dynamometer is obtained as the sensor mounting height changes, as shown in Figure 7. The theoretical value of *K* is obtained by the Equation (25), and the simulation value of *K* is obtained by the result of the finite element analysis.

Figure 7 shows that the maximum error between the simulation result of *K* and the theoretical result is 7.8%, and the trend of the two is generally consistent. Therefore, the applicability of the theoretical model of the dynamometer strain is proven by finite element simulation. When *h* = 20 mm, *K* obtains the maximum value, in other words, the dynamometer has the maximum measurement sensitivity. At the same time, the dynamometer’s maximum ultimate load is 1667 N and the bending stiffness is reduced by 7%.

**Figure 8:**
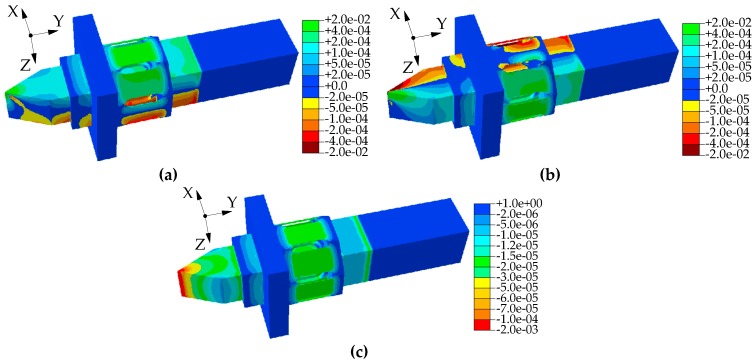
Dynamometer strain cloud: (**a**) Strain cloud with one-way force *F_z_* of 1000 N, (**b**) strain cloud with one-way force *F_x_* of 1000 N, (**c**) strain cloud with one-way force *F_y_* of 1000 N.

The static simulation of the dynamometer with *h* = 20 mm is performed using ABAQUS software. Figure 8 shows that the maximum strain of the dynamometer occurs at the center of the sensor substrate. The distribution of the strain cloud shows that the elastomer of the dynamometer has a relatively significant strain effect. Therefore, the structural design of the dynamometer is reasonable and superior. When the input voltage *U*_0_ of circuits 1–3 is 10 V, the maximum strain of the dynamometer is 4.89 × 10^−4^, which is a 4.5% difference from the theoretical value in Figure 8a. The measuring sensitivity of the dynamometer is 108.77 N/mV for *F_z_* using Equation (4). The maximum strain of the dynamometer is 4.85 × 10^−4^, which is a 5.3% difference from the theoretical value in Figure 8b. The measuring sensitivity of the dynamometer is 109.67 N/mV for *F_x_* using Equation (5). The maximum strain of the dynamometer is 1.73 × 10^−5^, which is a 4.8% difference from the theoretical value in Figure 8c. The measuring sensitivity of the dynamometer is 2934.18 N/mV for *F_y_* using Equation (6).

**Figure 9:**
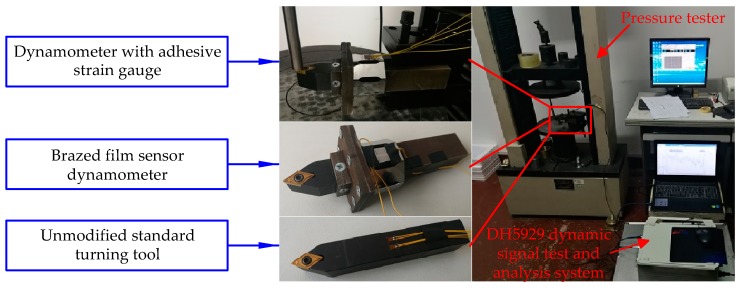
Dynamometer bending test.

In order to verify the good strain effect of the dynamometer, the dynamometer with a sensor mounting height of 20 mm is subjected to a bending test. In the experiment, the dynamometer with adhesive strain gauge, the dynamometer of the brazed film sensor, and the standard turning tool are compared, as shown in Figure 9. Among them, several strain resistor grids are connected to the DH5929 dynamic signal test and the analysis system to measure the strain value of the elastic substrate.

**Figure 10:**
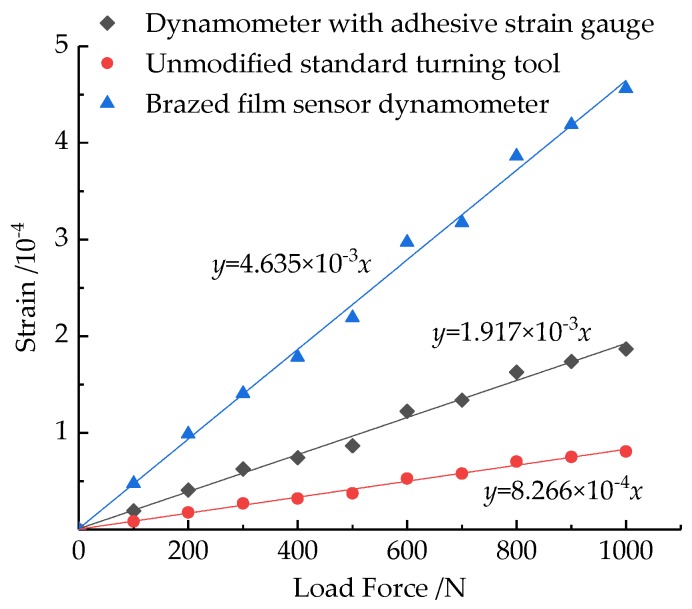
Relationship between substrate strain and applied load.

Figure 10 shows the relationship between the strain of the elastic substrate and the applied load when a one-way force *F_z_* from 0 to 1000 N is applied to the tool nose. The relationship between the two has a good linearity, and the slope of the fitted curve is *K*_1_. The *K*_1_ value of the brazed film sensor dynamometer is 4.635 × 10^−7^, which is a 8.6% difference from the theoretical value. The *K*_1_ value of the dynamometer with adhesive strain gauge is 1.917 × 10^−7^, which is a 62.2% difference from the theoretical value. Obviously, this is the result of the pasting process reducing the strain transmission efficiency. However, the *K*_1_ value of the dynamometer with adhesive strain gauge is about 2.32 times that of the standard turning tool, which is a 5.8% difference from the theoretical calculation result. Experiments show that the dynamometer can improve the strain sensitivity of the measurement system, which helps the measurement signal acquisition.

## 6. Conclusions

This paper presents a turning dynamometer embedded in a film strain sensor, which can measure three-way cutting forces and has high-strain sensitivity. By equating the dynamometer with a cantilever beam model and performing segmentation processing of equal sections, the relationship between the output voltage of the dynamometer system and the cutting force components is obtained, and the mathematical model of the dynamometer’s structural parameters affecting its strain sensitivity coefficient *K* is established. The structural dimensions of the standard turning tool with 20 mm square is used as a reference. When the mounting height of the film sensor is 20 mm, the strain sensitivity coefficient *K* of the dynamometer is the largest. The experiments show that the fabricated Nichrome film sensor has a good strain effect and is suitable for the dynamometer. The finite element analysis indicates that the dynamometer’s elastomer has good strain effect, and also verifies the applicability of the theoretical model of the dynamometer strain. Finally, the bending test of the dynamometer shows that the *K* value of the dynamometer with the adhesive strain gauge is 2.32 times that of the standard turning tool, which is a 5.8% difference from the theoretical value. The *K* value of the brazed film sensor dynamometer is 2.42 times that of the dynamometer with adhesive strain gauge, which indicates that the brazed film sensor dynamometer can effectively eliminate the influence of the pasting process on the strain transmission efficiency in the traditional dynamometer. The research in this paper reveals the great potential of this dynamometer and provides a good foundation for subsequent product applications.

## Figures and Tables

**Table 1:** Fabrication parameters and basic characteristics of the thin-film sensor.

	Material	Deposition Pressure	Power	Deposition Time	N_2_ Flow	Ar Flow	Roughness	Thickness
Substrate	8K mirror stainless steel	-	-	-	-	-	236 nm	0.3 µm
Insulating layer	Si_3_N_4_	1.2 Pa	120 W	360 min	10 sccm	80 sccm	231 nm	2.3 μm
Sensitive layer	Ni_80_Cr_20_	1.2 Pa	120 W	20 min	-	70 sccm	218 nm	0.8 μm
Protective layer	Si_3_N_4_	1.2 Pa	120 W	360 min	10 sccm	80 sccm	215 nm	2.3 μm
